# The role of interleukin-18 in glioblastoma pathology implies therapeutic potential of two old drugs—disulfiram and ritonavir

**DOI:** 10.1186/s40880-015-0010-1

**Published:** 2015-04-09

**Authors:** Richard E Kast

**Affiliations:** International Initiative for Accelerated Improvement of Glioblastoma Care Study Center, 22 Church Street, Burlington, VT 05401 USA

**Keywords:** Chemotherapy, Disulfiram, Glioblastoma, Interleukin-18, Migration, Ritonavir

## Abstract

Based on reporting in the last several years, an impressive but dismal list of cytotoxic chemotherapies that fail to prolong the median overall survival of patients with glioblastoma has prompted the development of treatment protocols designed to interfere with growth-facilitating signaling systems by using non-cytotoxic, non-oncology drugs. Recent recognition of the pro-mobility stimulus, interleukin-18, as a driver of centrifugal glioblastoma cell migration allows potential treatment adjuncts with disulfiram and ritonavir. Disulfiram and ritonavir are well-tolerated, non-cytotoxic, non-oncology chemotherapeutic drugs that are marketed for the treatment of alcoholism and human immunodeficiency virus (HIV) infection, respectively. Both drugs exhibit an interleukin-18–inhibiting function. Given the favorable tolerability profile of disulfiram and ritonavir, the unlikely drug-drug interaction with temozolomide, and the poor prognosis of glioblastoma, trials of addition of disulfiram and ritonavir to current standard initial treatment of glioblastoma would be warranted.

## Introduction

After diagnosis, the median overall survival of patients with glioblastoma is approximately 2 years [1]. The last advance in treatment was the introduction of the Stupp protocol in 2005, which involves maximal resection followed by temozolomide and irradiation [1,2]. Recurrence occurs in almost all treated patients [1]. Over 40 trials of various traditional cytotoxic cancer chemotherapeutic drugs reported in the last few years have failed to significantly improve outcomes for patients with glioblastoma [3,4].

The current paper was, in part, prompted by a paper by Yeh *et al*. [5] in 2012 that clearly noted the crucial role of interleukin-18 (IL-18) in driving the centrifugal migration of glioblastoma cells. Starting with a premise that “the mediators and cellular effectors of inflammation are important (growth-enhancing) constituents of the local environment of tumors,” Yeh *et al*. [5] showed that non-malignant brain resident microglia secreted increased active IL-18 when stimulated by a growing glioblastoma. The results of this study indicated that such triggering is via specific mediation of extracellular matrix proteins, particularly fibronectin and vitronectin synthesized and secreted by glioblastoma cells. This IL-18–stimulated centrifugal migration is an important link between glioblastoma pathology and treatment resistance [5,6]. If such data in support of a feed-forward cycle can be supported by further study, this would be a perfect example of “cancer cells communicating actively” with each other and with host cells and organs [7], giving us a tremendous insight into glioblastoma pathology with immediate treatment consequences, which this paper will delineate.

## Review

### IL-18 in glioblastoma

**Figure 1:**
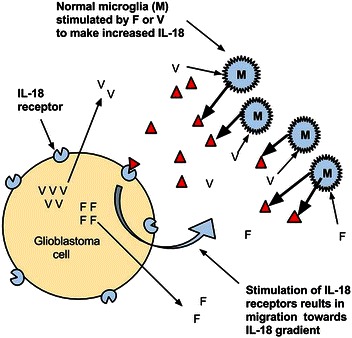
**Feed-forward interleukin-18 (IL-18) cycle.** Schematic summary of the growth-enhancing feed-forward cycle reported by Yeh *et al*. [5] shows that microglial-synthesized IL-18 (red triangles) facilitates the migration of glioblastoma and that glioblastoma-synthesized fibronectin (F) or vitronectin (V) stimulates microglial IL-18 synthesis.

In line with the observations by Yeh *et al*. [5], IL-18 has been identified as an important growth- and motility-driving element in many cancers (Figure 1). IL-18 is generally initially synthesized as a 24-kDa pro–IL-18 form, later proteolytically maturing to its active 18-kDa form. Both processes have been identified in a variety of cancers, such as gastric cancer [8], squamous cell carcinoma [9,10], pancreatic cancer [11], epithelial ovarian cancer [12], both primary and bone metastatic non–small cell lung cancer [13], prostate cancer [14], small cell lung cancer [15], hepatocellular carcinoma [16], metastatic melanoma [17], and other human cancers.

Mammalian brain astrocytes and microglia express both IL-18 and IL-18 receptors [18-22], forming an integral part of both normal glia-neuron dialogue and brain tissue response to injury. Normal brain resident microglia increase the synthesis of IL-18 under conditions of infection, hypoxic-ischemic, and traumatic brain injuries, for example [23,24]. IL-18 is an important link in the development of both normal protective and pathological inflammation [25,26], among many other pathways, by promotion of interferon gamma synthesis and furthering Th1 helper T-cell development [27]. IL-18 is a core mediator of angiogenesis and inflammation in rheumatoid arthritis pannus formation [28].

Proteolytic maturation is mediated by IL-1beta–converting enzyme (ICE), synonymous with caspase-1. Caspase-1 is a 60-kDa multimeric protease composed of two 20-kDa and two 10-kDa subunits. Caspase-1 itself is translated initially into a 45-kDa pro-caspase-1, appearing on the outer cell membrane [29,30].

### IL-18 and cellular migration

Triggering migration is among the more prominent effects of exposing cells to IL-18. The migratory capacity of normal cardiac fibroblasts [31,32], normal macrophages [33], vessel wall transmigrating neutrophils [34], and coronary artery smooth muscle cells [35] increases in response to IL-18 exposure. Migration rate of normal human skin melanocytes increases after IL-18 exposure [36], as does that of dermal Langerhans cells [37] and murine melanoma cells [38,39].

IL-18–mediated increase in centrifugal migration was observed in gastric cancer cells [8,40] similar to what Yeh *et al*. [5] found in glioblastoma. Increased circulating IL-18 was observed in patients with gastric cancer [41], head and neck squamous cell carcinoma [10], esophageal cancer [41], epithelial ovarian cancer [42], and non–small cell lung cancer [15,43] where higher levels of IL-18 were associated with poorer overall survival [13], in patients with breast cancer [44] where levels of IL-18 in metastatic disease were also higher than those in non-metastatic disease [45], and in patients with prostate cancer [14], small cell lung cancer [15], and pancreatic cancer where higher levels of IL-18 predicted poorer overall survival [46]. The common theme in these studies is that IL-18 increases most with metastatic disease. These findings, combined with IL-18–stimulated centrifugal migration in gastric cancer cells [8,40] and glioblastoma cells [5], point to IL-18 as a general mobility-enhancing signaling molecule for cancers.

A dramatic and instructive finding in this regard was reported by Jiang *et al*. [47] in 2003. In studying two subclones of the same human lung cancer cell line, one highly metastatic and the other poorly so, the authors concluded that robust IL-18 synthesis by the metastatic subclone was the determining factor in the different subclones’ behaviors, namely higher motility and metastatic competence in the higher IL-18–producing subclone.

### Disulfiram and ritonavir

Disulfiram is a 297-Da aldehyde dehydrogenase inhibitor used clinically since the 1950s to treat alcoholism, and it is still widely used worldwide [48]. Ritonavir is a 721-Da antiviral drug, one of the first-generation protease inhibitors marketed in the 1980s to treat human immunodeficiency virus (HIV) infection [49].

Disulfiram and ritonavir form 2 of the 9-drug regimen to augment temozolomide in the coordinated undermining of survival paths 9 (CUSP9*) treatment protocol for recurrent glioblastoma. The rationale for these drugs was provided in the CUSP9 and CUSP9* papers [3,4], but it did not include considerations of the effects of disulfiram or ritonavir on IL-18. Detailed pharmacologic analysis in the CUSP9 papers indicated the unlikelihood of drug-drug interaction between either disulfiram or ritonavir or both and temozolomide [3,4]. What not discussed in these papers but reviewed here are additional data indicating that disulfiram and ritonavir can limit the maturation of pro–IL-18 and therefore be useful during primary Stupp protocol treatment.

The data showing the function of IL-18 in inhibiting the actions of disulfiram and ritonavir were previously reviewed in connection with their potential to mitigate inflammation associated with acute pancreatitis [50] or central nervous system (CNS) inflammation after blast exposure [51]. A study reported in 1997 showed potent caspase-1 inhibition by disulfiram [52], which would block pro–IL-18 maturation. Ritonavir decreases caspase-1 expression [53-55].

We have convincing evidence that both ritonavir and disulfiram or their active metabolites penetrate the blood–brain barrier effectively in sufficient concentrations [56-60]. The most common clinical use of disulfiram is to inhibit aldehyde dehydrogenase during the treatment of alcoholism [48]. A secondary use of disulfiram is to inhibit brain dopamine beta-hydroxylase during the treatment of certain addictions [56,57], thus indicating sufficient blood–brain penetration. The levels of ritonavir in the brain tissues and cerebrospinal fluid (CSF) tend to be low [58] when given orally, but can easily be increased from 2.4 to 6.6 ng/mL CSF with oral co-administration of ketoconazole [59]. We believe that these levels are sufficient based on *in vitro* studies and observations of CSF clearance of HIV with oral ritonavir [60].

### Additional IL-18 considerations

Exogenous IL-18 is being investigated in several active research programs for its ability to stimulate immune responses to glioma cells [61-63]. Data indicating that IL-18 can enhance an anti-tumor immune response as well as being a trophic factor for many cancers were reviewed in 2007 by Park *et al*. [64]. Which factors predominate during human cancer progression remains unclear today. Given 1) the findings of Yeh *et al*. [5], which are concordant with a wealth of data on the active role of IL-18 in the dissemination of other cancers, and 2) the widely dispersed microsatellites within the brain tissues that go on to be fatal in glioblastoma, the safest supposition for now is that the net effect of IL-18 in glioblastoma is negative.

Thus, there is potential for the suggested combination of disulfiram and ritonavir to reduce an immune response to glioblastoma cells, but if the preponderant effect of IL-18 is to stimulate centrifugal migration, the net effect may well be clinically beneficial. Given the theoretical immunostimulatory aspect of IL-18 function, the paradox of finding increased circulating IL-18 as a negative prognostic portent has been discussed without resolution in the context of both pancreatic cancer [65] and breast cancer [66].

The 9-drug regimen CUSP9* designed for recurrent glioblastoma after Stupp protocol treatment [3] already includes both disulfiram and ritonavir for reasons that do not include IL-18 inhibition. Given the likely centrifugal migration driven by IL-18 and unlikely additional adverse effect burden of adding concurrent disulfiram and ritonavir, we have the potential to improve initial treatment with the Stupp protocol [1,2], temozolomide, and irradiation after maximal surgical resection.

*In vitro* irradiation of microglia increases their IL-18 synthesis [67]. *In vivo* brain irradiation up-regulates microglial IL-18 synthesis *in situ* and increases the number of IL-18–producing microglia [68]. Of particular note, irradiation-induced increased microglial IL-18 is not transient and may indeed be permanent [68]. Indeed, low-dose, whole body irradiation dose proportionately increases circulating IL-18 in mice, pigs, and non-human primates [69]. Thus, as part of the standard Stupp protocol for initial treatment of glioblastoma, in addition to killing much of the primary tumor mass and consequently somewhat lengthening overall survival, irradiation can be expected to stimulate IL-18–driven centrifugal migration of the few surviving glioblastoma cells. Stimulating the centrifugal migration of glioblastoma cells leads to their wide dissemination and sets up conditions for later fatal recurrence, the classic double-edged sword. Disulfiram and ritonavir may have potential to overcome the pathophysiology of glioblastoma and improve the results of the Stupp protocol as currently constituted.

## Conclusions

We have demonstrated how a feed-forward, IL-18–based, growth-enhancing system forms an element of glioblastoma pathophysiology whereby glioblastoma cells secrete extracellular matrix proteins, such as fibronectin and vitronectin, and these proteins then stimulate surrounding normal brain microglia to secrete increased IL-18 [5]. The accumulation of IL-18 stimulates centrifugal glioblastoma cell migration and then stimulates a new set of microglia at the growing front to synthesize IL-18. These centrifugally migrating cells ultimately prove to be untreatable and fatal.

Two old, well-tolerated, low-risk drugs, disulfiram and ritonavir, have been shown to interfere with IL-18 generation/function, but with little evidence that they would increase the burden of adverse effects or interfere with Stupp protocol interventions, temozolomide, and radiation. Therefore, the risk-benefit ratio favors adding concomitant disulfiram and ritonavir to the standard Stupp protocol.
